# On the *Domene* species of China, with descriptions of four new species (Coleoptera, Staphylinidae)

**DOI:** 10.3897/zookeys.456.8413

**Published:** 2014-11-21

**Authors:** Benedikt Feldmann, Zhong Peng, Li-Zhen Li

**Affiliations:** 1 Juistweg 1, 48159 Münster, Germany; 2 Department of Biology, College of Life and Environmental Sciences, Shanghai Normal University, Shanghai, 200234, P. R. China

**Keywords:** Coleoptera, Staphylinidae, Paederinae, *Domene*, Palaearctic region, China, new species, lectotype designation, neotype designation, new synonymy, additional records, key to species

## Abstract

Material of the paederine genus *Domene* Fauvel, 1873 from China is examined. Nine species were identified, four of them described previously, one unnamed (represented exclusively by females), and four are newly described: *Domene cultrata* **sp. n.** (Gansu, Hubei, Shaanxi); *Domene cuspidata* **sp. n.** (Gansu, Shaanxi, Sichuan); *Domene chenae* **sp. n.** (Guangxi); *Domene reducta* **sp. n.** (Sichuan). A lectotype is designated for *Domene reitteri* Koch, 1939; a neotype is designated for *Domene chenpengi* Li, 1990. *Domene dersuuzalai* Gusarov, 1992 is placed in synonymy with *Domene chenpengi*. Previous records of two Japanese species from China are most likely based on misidentifications and considered erroneous. Thus, the *Domene* fauna of China is currently composed of twelve described species. A key to the *Domene* species of China is provided. The distributions of eleven species are mapped.

## Introduction

In contrast to the West Palaearctic *Domene* fauna, which can be considered rather well-studied, the known inventory of the East Palaearctic and Oriental faunas, which have received less taxonomic attention, is still incomplete. Prior to the present study, eleven species had been recorded from China, including Taiwan, three of them very recently: *Domene alesiana* Assing & Feldmann, 2014 (Taiwan); *Domene chenpengi* Li, 1990 (Jilin); *Domene crassicornis* (Sharp, 1874) (Jilin); *Domene curtipennis* Sharp, 1889 (Liaoning); *Domene dersuuzalai* Gusarov, 1992 (Beijing); *Domene firmicornis* Assing & Feldmann, 2014 (Zhejiang); *Domene immarginata* Assing & Feldmann, 2014 (Yunnan); *Domene malaisei* Scheerpeltz, 1965 (Yunnan); *Domene procera* Eppelsheim, 1886 (Northeast Territory); *Domene reitteri* Koch, 1939 (Zhejiang), and *Domene scabripennis* Rougemont, 1995 (Taiwan) (Eppelsheim 1886; Koch 1939; Scheerpeltz 1965; Coiffait 1982; Li et al. 1990; Li 1992; Gusarov 1992; Rougemont 1995; Smetana 2004; Assing and Feldmann 2014). Except for *Domene chenpengi*, which is listed as incertae sedis by Smetana (2004), all the Chinese *Domene* species have been attributed to the subgenus *Macromene* Coiffait; for a comment on the subgeneric concept of *Domene* currently in use see Assing and Feldmann (2014).

In recent years we obtained numerous *Domene* specimens from several public and private collections. Nine species were identified, four of which are described for the first time.

## Material and methods

The examined material is deposited in the following public and private collections:

HNHM Hungarian Natural History Museum, Budapest (Gy. Makranczy)

NHMB Naturhistorisches Museum, Basel (M. Geiser, I. Zürcher)

NHMW Naturhistorisches Museum Wien (H. Schillhammer)

MNHUB Museum für Naturkunde der Humboldt-Universität, Berlin (J. Frisch)

SNUC Insect Collection of Shanghai Normal University, Shanghai

RMS Riksmuseum, Stockholm (B. Viklund)

cAss private collection Volker Assing, Hannover

cFel private collection Benedikt Feldmann, Münster

cPüt private collection Andreas Pütz, Eisenhüttenstadt

cRou private collection Guillaume de Rougemont, Oxford

cSch private collection Michael Schülke, Berlin

cSme private collection Aleš Smetana, Ottawa

The genitalia and other dissected parts were mounted on plastic slides and attached to the same pin as the respective specimens. Photographs were taken with a Canon EOS 7D camera with a MP-E 65 mm macro lens or with a Canon G9 camera mounted on an Olympus CX31 microscope. The map is created using MapCreator 2.0 (primap) software.

The following abbreviations are used in the text, with all measurements in millimeters:

Total length (TL): length of body from anterior margin of mandibles (in resting position) to abdominal apex.

Length of forebody (FL): length of forebody from anterior margin of mandibles to posterior margin of elytra.

Head length (HL): length of head from anterior margin of frons to posterior constriction of head.

Head width (HW): maximum width of head.

Length of antenna (AnL).

Neck width (NW): maximum width of neck.

Length of pronotum (PL).

Width of pronotum (PW).

Elytral length (EL): length at suture from apex of scutellum to elytral hind margin.

Elytral width (EW): combined width of elytra.

Length of metatibia (TiL).

Length of metatarsus (TaL).

Width of segment VI (AW).

Length of aedeagus from apex of ventral process to base of aedeagal capsule (AL).

The type labels are cited in the original spelling; different labels are separated by slashes.

## Results

Thirteen *Domene* species, ten of them exclusive and one of them unnamed, are known from China (including Taiwan). Four species are described for the first time, a new synonymy is proposed and two species are deleted from the list of Chinese *Domene* species.

Based on the male sexual characters, mainly the shape and chaetotaxy of sternite VIII and the morphology of the aedeagus, as well as on external characters such as the punctation and sculpture of the head, pronotum and elytra, the Chinese representatives of *Domene* are attributed to five different species groups.

The *Domene scabripennis* group: see Assing and Feldmann (2014). Note that the placement of *Domene firmicornis* in this group is doubtful. Neither the male nor the female sexual characters suggest closer phylogenetic affiliations to any of the other species known from China.

The *Domene malaisei* group comprises four species (*Domene malaisei*, *Domene cultrata*, *Domene cuspidata*, *Domene reducta*) distributed in the midwest and southwest of China. They share the following differential characters: large body size (length of forebody 4.70–5.50 mm); head and pronotum with moderately coarse and dense punctation; pronotum relatively large and oblong; protarsomeres I–IV weakly dilated in both sexes; elytra with moderately coarse, not coriaceous and irregular macropunctation, with additional micropunctation, without distinct longitudinal elevations and without pronounced impressions; male sternite VII with modified short, stout, black setae; sternite VIII with shallow median impression, this impression with strongly modified, stout black setae, on either side of the deep and almost V-shaped posterior excision with a dense cluster of black setae; ventral process of aedeagus (in lateral view) not conspicuously slender, rather stout.

The *Domene reitteri* group includes two species (*Domene reitteri*, *Domene chenae*) distributed in the east and south of China and is distinguished by the following character combination: moderately large body size (length of forebody 4.16–4.73 mm); head and pronotum with fine and dense punctation; pronotum large and moderately oblong; protarsomeres I–IV weakly dilated in both sexes; elytra without rough surface, with fine, dense and uniform punctation; male sternite VII with moderately to strongly modified short, stout, black setae; sternite VIII with shallow median impression, this impression with strongly modified stout black setae, on either side of the moderately deep and U-shaped posterior excision without cluster of setae; ventral process of aedeagus (in lateral view) relatively stout.

*Domene chenpengi* and *Domene procera* belong to two different species groups which comprise additional species from Japan. *Domene chenpengi* is closely related to *Domene curtipennis* and allied species, *Domene procera* is closely related to *Domene crassicornis* and allied species. A detailed characterization of these species groups requires a revision of the *Domene* fauna of Japan.

### Domene (Macromene) chenpengi

Taxon classificationAnimaliaColeopteraStaphylinidae

Li et al., 1990

Figs 1 , 2A , 3

Domene chenpengi Li et al., 1990: 66.
Domene (Macromene) dersuuzalai Gusarov, 1992: 21; syn. n.

#### Type material.

Neotype ♂, present designation: “China: Beijing, ca. 1400 m, Dongling Mts, 15.Vi.2001, Xiaolongmen, Liu Lang Yu / N39°97, E115°43 [sic], Mixed woodland litter, Leg. J. Cooter + P. Hlavá [sic] / Neotypus ♂ *Domene chenpengi* Li desig. B. Feldmann & Z. Peng 2014 / Domene chenpengi Li, det. B. Feldmann 2014“ (MNHUB).

#### Comment

The original description is based on a single male specimen from Chang Chun [ca. 43°45'N, 125°27'E], Jing Yue, collected on 30.VII.1985 by Peng Chen (Li et al. 1990). Inquiries into the whereabouts of the holotype at the Northeast Normal University, where the holotype should be deposited, yielded no results. It was looked for in the respective collection, but not found (personal communication Xiu-Qing Yin, one of the authors in Li et al. 1990 and director of the biogeographical office of Northeast Normal University, e-mail 5 May, 2014; personal communication Jing-Ke Li, author of *Domene chenpengi* and guest professor of the Harbin Normal University, e-mail 5 May, 2014). Thus, the type specimen must be regarded as lost. The insufficient description of *Domene chenpengi*, which fails to provide any illustration whatsoever, is in agreement with examined material previously identified as *Domene dersuuzalai* from the Russian Far East and China, particularly regarding the habitus and the characteristic shape of the male sternite VIII with its shallow posterior excision. Moreover, the type locality of *Domene chenpengi* accords with the known distribution of *Domene dersuuzalai*. In the interest of stability of nomenclature, a neotype designation is deemed necessary to stabilize the present interpretation of *Domene chenpengi* and the synonymy with *Domene dersuuzalai*. To this end, a male from the Dongling mountains in Beijing, a locality reasonably close to the type locality, is designated as the neotype. Based on the detailed description of *Domene dersuuzalai* (Gusarov 1992), the species is doubtlessly conspecific with the neotype of *Domene chenpengi*; hence the synonymy proposed above.

#### Material examined

**(60 exs.). Russian Far East: Primorskiy Kray:** 2 exs., Vladivostok env., Sedanka, 28.VII.1992, leg. Beloborodov (NHMB, cFel); 3 exs., Vladivostok, 11.VII.1993, leg. Pütz & Wrase (cSch); 6 exs., N Vladivostok, “Seitengraben des Parwaja Rjetschka Tales”, 1918–1920, leg. Frieb (NHMW, cFel); 1 ex., Kamenushka, 14.–15.VII.1992, leg. Beloborodov (NHMB); 7 exs., Partisansky district, Alexeyevskiy khrebet, 20 km E Sergeyevka, forests near Andreyevka river, 400–800 m, 26.–29.VII.1993, leg. Pütz & Wrase (cSch, cFel); 1 ex., S Artyom town, Ozernyy Kluytch river, 100–300 m, 10.V.–5.VI.2002, leg. Plutenko (cSch); 2 exs., Lazovskiy reserve, 9 km SE Kievka, lodge Petrova env., 3.–8.VI.1994, leg. Sundukov (cPüt); 1 ex., same data, but 9.–13.VI.1995 (cPüt); 1 ex., Lazovskiy district, Kovarinovo, 5 km NE Lazo, spring valley, 1.–5.VI.1995, leg. Sundukov (cFel); 1 ex., Lazovskiy reserve, Kordon Amerika, 134°03'01"E, 43°16'16"N, 14.–17.V.1999, leg. Sundukov (cSch); 2 exs., Lazovskiy reserve, Kordon Amerika, 18.–19.VI.1997, leg. Sundukov (cSch); 1 ex., Lazovskiy reserve, Kordon Petrova, 133°47'55"E, 42°52'14"N, 19.–20.IX.1999, leg. Sundukov (cSch); 1 ex., Lazovskiy reserve, Kordon Proselochny, 134°07'43"E, 43°00'34"N, 4.–6.X.1999, leg. Sundukov (cSch); 3 exs., Sikhote-Alin reserve, Jasnaya estuary, 26.VI.–4.VII.1998, leg. Sundukov (cAss, cSch); 1 ex., Siniy khrebet, 4 km E Evseevki, 7.–9.VIII.1999, leg. Shavrin (cSch); 1♂, 2 ♀♀ [identified by Gusarov 1995 as *Domene dersuuzalai*], Arsenev env., 27.V.–5.VII.1991, leg. Sausa (NHMW). **Khabarovskiy Kray:** 3 exs., SE Boitsovo, 12 km NE Bikin, 250–350 m, 26.V.–4.VI.1990, leg. Schawaller (cSch). **Sakhalin:** 5 exs., Moneron Island, 15.VI.–6.VII.2002, leg. Plutenko (cSch, cFel). **China: Beijing:** 1 ♂, 5 ♀♀, Xiaolongmen, Yan Shan, Dongling Mts, 1400 m, 15.–16.VI.2001, leg. Hlavač & Cooter (cAss, cSch, cFel); 2 ♂♂, Xiaolongmen, Liu Lang Yu, Dongling Mts, 39°58'N, 115°26'E, ca. 1400 m, under fungoid *Juglans* bark, 15.VI.2001, leg. Cooter & Hlavač (cRou); 1 ♀, Xiaolongmen, Liu Lang Yu, Dongling Mts, 39°58'N, 115°26'E, ca. 1400 m, mixed forest litter, 15.VI.2001, leg. Cooter & Hlavač (cRou); 2 ♂♂, 1 ♀, Xiaolongmen, Mei Yao Yu, Dongling Mts, 39°58'N, 115°26'E, ca. 1400 m, mixed forest litter, 16.VI.2001, leg. Cooter & Hlavač (cRou, cFel); 1 ♂, 2 ♀♀, Miyun County, Wulin Shan, 40°36'N, 117°23'E, 750–850 m, 8.–9.VII.2006, leg. Shen & Tang (SNUC). **South Korea:** 1 ♂, Gangwon-do, Seorak-san, 1.5 km S Han-gyeryeong pass rest station, roadside forest, 38°05'26"N, 128°24'03"E, 790 m, from wet, fungusy leaf litter, under trunk, rocks, sifted, 9.IX.2010, leg. Makranczy & al. (HNHM).

#### Redescription.

Measurements (in mm) and ratios: TL 7.15–7.60, FL 3.80–3.95, HL 1.1–1.15, HW 1.00–1.08, AnL 2.55–2.65, NW 0.35–0.38, PL 1.19–1.23, PW 0.95–1.03, EL 0.98–1.03, EW 1.03–1.05, TiL 1.20–1.25, TaL 0.90–0.98, AW 1.08–1.13, AL 0.85–0.90, HL/HW 1.05–1.13, HW/PW 1.02–1.08, HL/PL 0.90–0.95, NW/HW 0.35–0.36, PL/PW 1.17–1.25, EL/PL 0.82–0.85.

Habitus as in Fig. 2A. Head and pronotum blackish brown; elytra brownish with anterior and posterior portions more or less extensively reddish brown; abdomen brownish; legs yellowish brown, except for the slightly paler tarsi; antennae light brown to yellowish brown.

**Figure 1.**
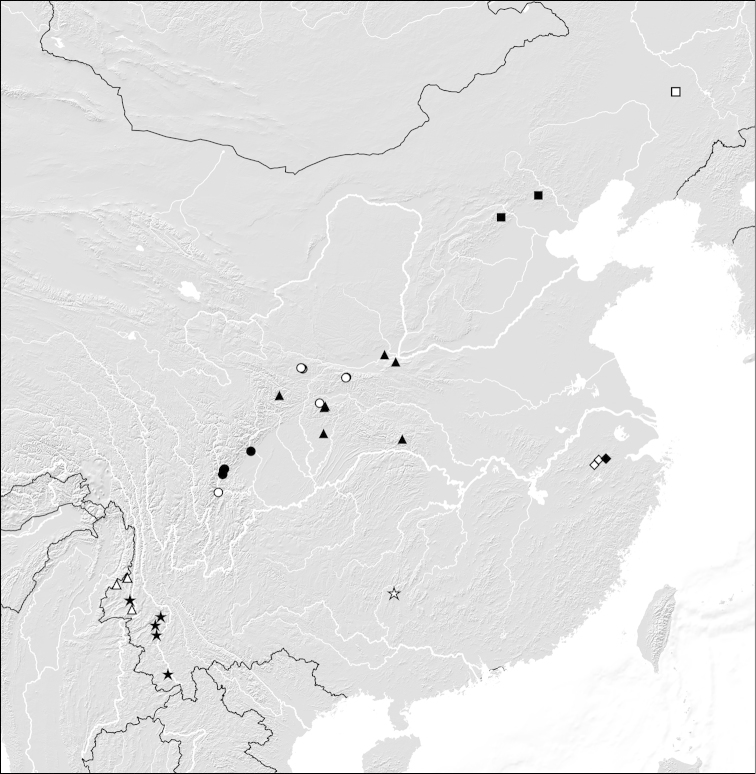
Distributions of *Domene* species in China: *Domene chenpengi* (filled squares; type locality: open square); *Domene firmicornis* (filled and open diamonds); *Domene immarginata* (filled stars); *Domene malaisei* (open triangles); *Domene reitteri* (filled diamond); *Domene chenae* (open star); *Domene cultrata* (filled triangles); *Domene cuspidata* (open circles); *Domene reducta* (filled circles).

**Figure 2.**
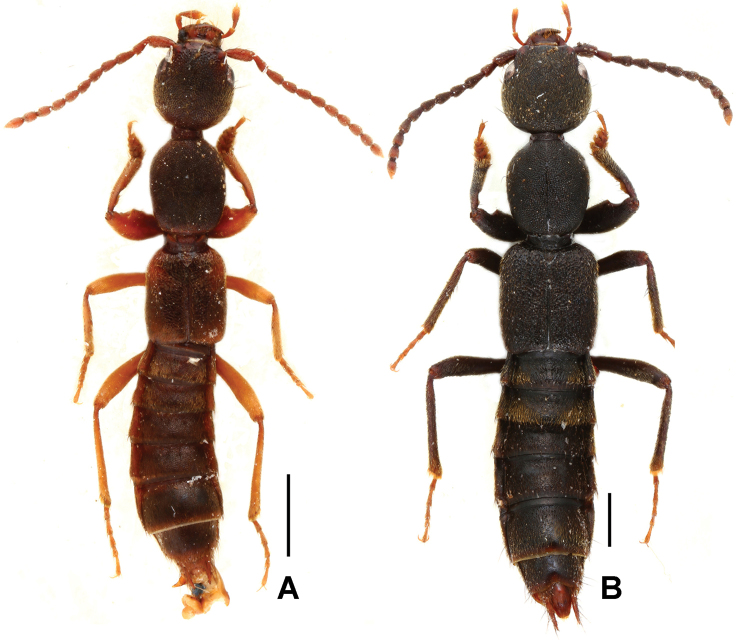
Habitus of *Domene* spp., **A** *Domene chenpengi* **B** *Domene firmicornis*. Scales: 1.0 mm.

**Figure 3.**
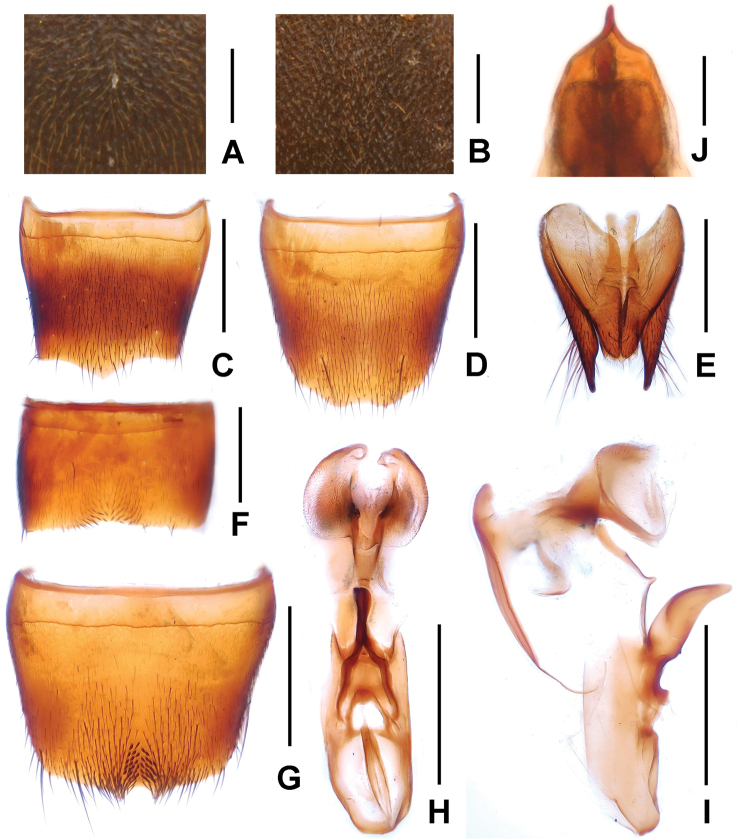
*Domene chenpengi*. **A** median dorsal portion of head **B** median portion of pronotum **C** female tergite VIII **D** female sternite VIII **E** female tergites IX–X. **F** male sternite VII **G** male sternite VIII **H** aedeagus in ventral view **I** aedeagus in lateral view **J** aedeagus in dorsal view. Scales: **A**–**B, J** 0.2 mm; **C**–**I** 0.5 mm.

Head orbicular, widest behind eyes; punctation (Fig. 3A) fine and very dense. All antennomeres longer than broad; antennomeres IV–X of equal length; antennomere I 1.9 times, II 1.2 times, III 1.3 times, XI 1.3 times as long as IV. Maxillary palpus slender, preapical joint about 2.5 times as long as broad.

Pronotum about as broad as head, widest in anterior third; lateral margins slightly convex in dorsal view; punctation (Fig. 3B) very fine with interstices forming narrow, longitudinal ridges.

Elytra without distinct longitudinal ridges; disc often more or less impressed; suture elevated in posterior three-fourths; macropunctation coarse, more or less dense and irregular on disc, interstices with shallow and irregular micropunctation; in lateral and posterior portions with distinctly finer and denser punctation. Hind wings present. Protarsomeres I–IV moderately dilated.

Abdomen with fine and very dense punctation on tergites III–VIII; interstices with microreticulation; tergite VIII more or less obtusely triangularly produced posteriorly (Fig. 3C); posterior margin of tergite VII with palisade fringe.

Male. Sternites III–VI unmodified; sternite VII (Fig. 3F) distinctly transverse, with shallow postero-median impression, this impression with a few modified short and black setae posteriorly; sternite VIII (Fig. 3G) transverse, with pronounced impression posteriorly, this impression with distinctly modified short and stout black setae, posterior excision very small; aedeagus as in Figs 3H–J, ventral process stout and apically moderately acute in lateral view; apical portion of dorsal fig long and distinctly sclerotized, basal portion short.

Female. Posterior margin of sternite VIII (Fig. 3D) in the middle with shallow concave excision; genital segments with distinctly sclerotized structure (Fig. 3E).

#### Comparative notes.

The similar external morphology, the similar chaetotaxy and shape of the male sternites VII and VIII, and especially the similar shape of the ventral process of the aedeagus suggest that *Domene chenpengi* is closely allied to *Domene curtipennis* from Japan. For illustrations of *Domene curtipennis* see Gusarov (1992: figure 4). Besides its conspicuous male sexual characters, *Domene chenpengi* is distinguished from the Chinese species of the *Domene malaisei* and *Domene scabripennis* groups and from *Domene procera* by its smaller size alone, and from species of the *Domene reitteri* group by its coarser and less densely punctate the elytra.

#### Distribution and natural history.

The currently known distribution ranges from the Russian Far East and Northern China (Beijing, Jilin) to South Korea. The specimens were partly sifted from leaf litter in mixed forest habitats or found under bark and rocks. The elevations range from 100 up to 1400 m.

### Domene (Macromene) crassicornis

Taxon classificationAnimaliaColeopteraStaphylinidae

(Sharp, 1874)

Lathrobium crassicornis Sharp, 1874: 59.

#### Comment.

*Domene crassicornis* was recorded by Li et al. (1990) from Jilin, the only record of this species from China. This record is evidently based on a misidentification and probably refers to *Domene procera*. Based on available evidence, the distribution of *Domene crassicornis* is restricted to Japan and consequently does not include China. All revised material from the Russian Far East belongs to *Domene procera*.

### Domene (Macromene) curtipennis

Taxon classificationAnimaliaColeopteraStaphylinidae

Sharp, 1889

Domene curtipennis Sharp, 1889: 261.

#### Comment.

The sole record of *Domene curtipennis* from China is that by Li (1992) from Liaoning. It is almost certainly based on a misidentification. Based on available evidence, the distribution of *Domene curtipennis* is restricted to Japan. All the examined material from the Russian Far East, South Korea and China belongs to *Domene chenpengi*, suggesting that *Domene curtipennis* does not occur in China.

### Domene (Macromene) firmicornis

Taxon classificationAnimaliaColeopteraStaphylinidae

Assing & Feldmann, 2014

Figs 1 , 2B , 4

Domene (Macromene) firmicornis Assing & Feldmann, 2014: 510.

#### Comment.

**Figure 4.**
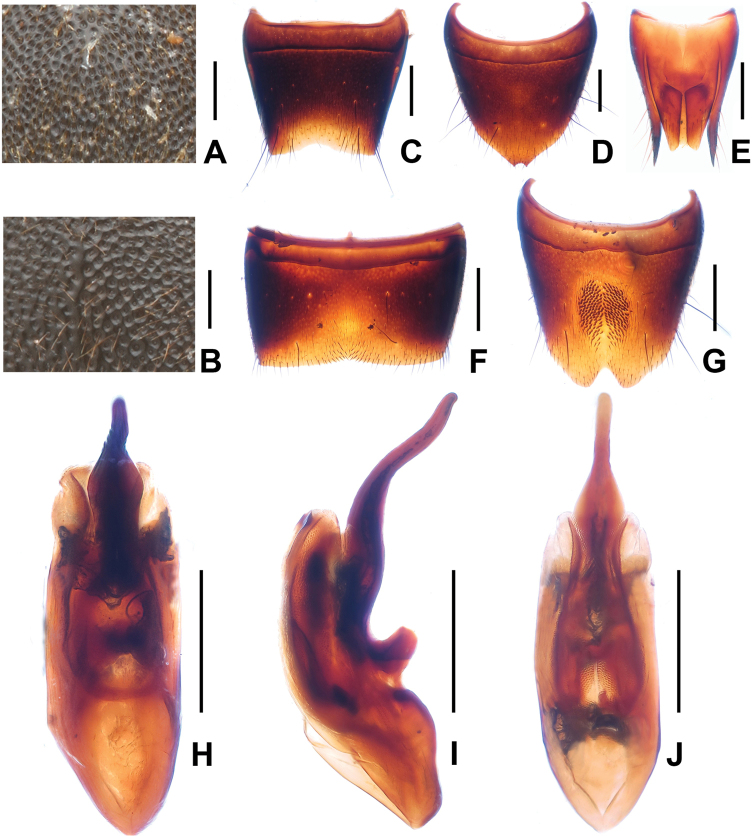
*Domene firmicornis*. **A** median dorsal portion of head **B** median portion of pronotum **C** female tergite VIIwI **D** female sternite VIII **E** female tergites IX–X. **F** male sternite VII **G** male sternite VIII **H** aedeagus in ventral view **I** aedeagus in lateral view **J** aedeagus in dorsal view. Scales: **A**–**B** 0.2 mm; **C**–**J** 0.5 mm.

Examined type specimens of this species are listed in an addendum in Assing and Feldmann (2014). The previously undescribed female sexual characters are as follows: female tergite VIII (Fig. 4C) with shallow postero-median impression and distinctly concave posterior excision; female sternite VIII (Fig. 4D) about as long as broad, posterior margin concave in the middle; sclerotized structure in female genital segments (Fig. 4E) symmetric and very weakly sclerotized. For illustrations of *Domene firmicornis* see Figs 2B, 4 and Assing and Feldmann (2014: figures 36–43).

### Domene (Macromene) malaisei

Taxon classificationAnimaliaColeopteraStaphylinidae

Scheerpeltz, 1965

Figs 1 , 5 , 6

Domene (Macromene) malaisei Scheerpeltz, 1965: 187.

#### Type material examined.

Holotype ♀: “N. E. Burma, Kambaiti, 2000 m, 4/6.1934, Malaise / HOLOTYPUS [red label] / TYPUS Domene malaisei O. Scheerpeltz [red label] / Domene malaisei nov. spec. det. Scheerpeltz, 1941 / 3884 E91” (RMS).

#### Additional material examined

(5 ♂♂, 9 ♀♀)**. China: Yunnan:** 4 ♂♂, 5 ♀♀, Tengchong County, Mingguang, Zizhi, Donghe, 25°42'N, 98°34'E, 2100–2300 m, 01.V.2013, leg. Peng & Song (SNUC, cAss); 4 ♀♀, same data, but 25°42'N, 98°35'E, 2500 m, 30.IV.2013 (SNUC); 1 ♂, Dehong Dai Autonomous Prefecture, mountain range 31 km E Luxi, 24°29'31"N, 98°52'58"E, 2280 m, secondary pine forest with old deciduous trees, litter sifted, 3.VI.2007, leg. Pütz (cFel).

#### Redescription.

Measurements (in mm) and ratios: Holotype: TL 8.90, FL 5.20, HL 1.38, HW 1.30, PL 1.45, PW 1.25, EL 1.50, HL/HW 0.94, HW/PW 1.10, HL/PL 0.89, PL/PW 1.16, EL/PL 1.03. Additional material: TL 7.90–9.20, FL 4.70–5.05, HL 1.24–1.33, HW 1.17–1.25, AnL 3.17–3.40, NW 0.40–0.46, PL 1.35–1.50, PW 1.15–1.25, EL 1.28–1.45, EW 1.44–1.53, TiL 1.57–1.65, TaL 1.14–1.33, AW 1.26–1.34, AL 1.07–1.18 HL/HW 1.06–1.07, HW/PW 0.99–1.01, HL/PL 0.89–0.93, NW/HW 0.34–0.38, PL/PW 1.15–1.20, EL/PL 0.95–0.97.

**Figure 5.**
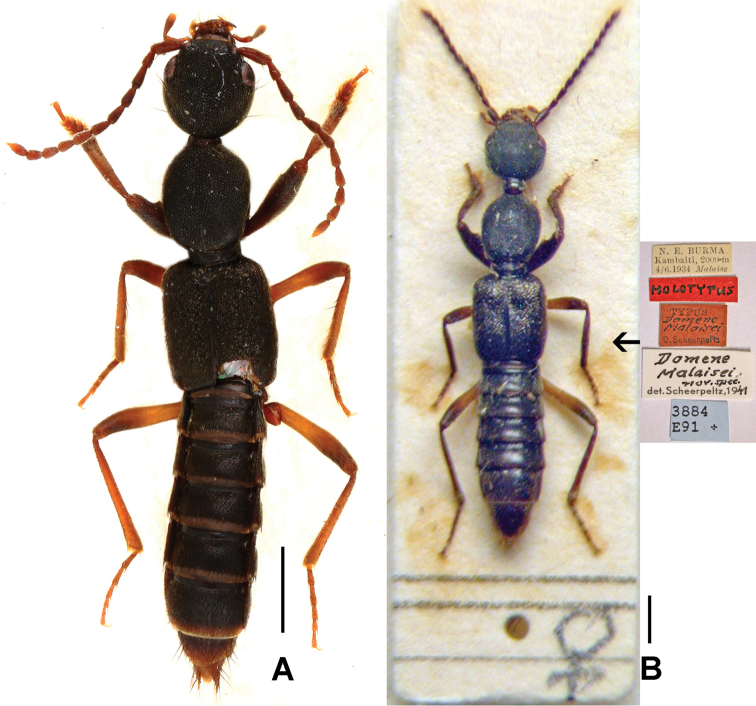
Habitus of *Domene malaisei*. **A** male **B** holotype. Scales: 0.5 mm.

Habitus as in Fig. 5. Body black; legs with blackish brown profemora and brown protibiae, basal halves of metafemora light brown, distal halves gradually infuscate; antennae dark brown to brown.

**Figure 6.**
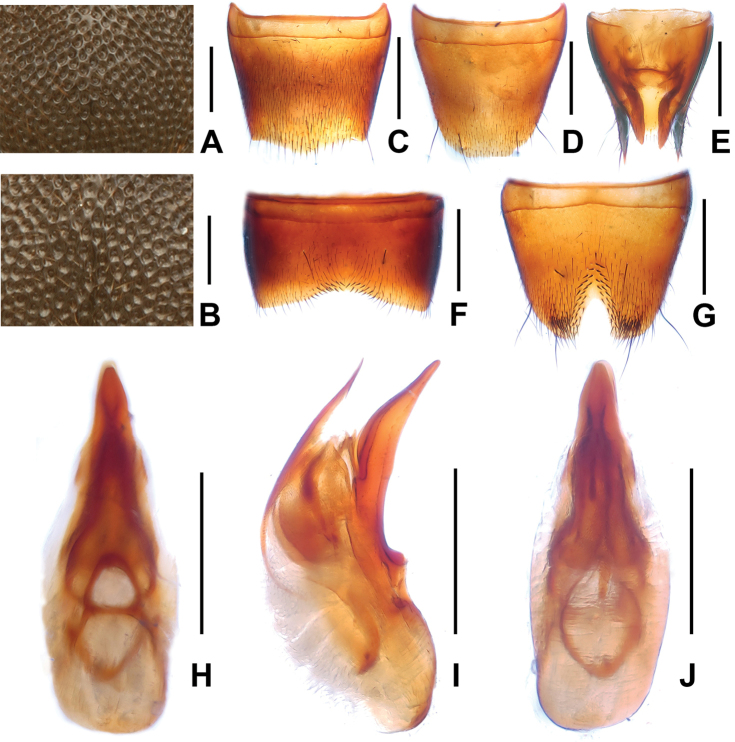
*Domene malaisei*. **A** median dorsal portion of head **B** median portion of pronotum **C** female tergite VIII **D** female sternite VIII **E** female tergites IX–X. **F** male sternite VII **G** male sternite VIII **H** aedeagus in ventral view **I** aedeagus in lateral view **J** aedeagus in dorsal view. Scales: **A**–**B** 0.2 mm; **C**–**J** 0.5 mm.

Head orbicular, broadest across eyes; punctation (Fig. 6A) coarse, distinctly umbilicate, and very dense, interstices forming very narrow ridges. All antennomeres longer than broad; antennomeres IV–X of equal length; antennomere I 1.3 times, II 0.9 times, III 1.1 times, XI 1.1 times as long as IV. Maxillary palpus very slender, preapical joint 2.8–3.1 times as long as broad.

Pronotum nearly as broad as head, widest in the middle; lateral margins convex in dorsal view; punctation (Fig. 6B) somewhat coarser than that of head; midline with rudiment of fine glossy line.

Elytra without distinct longitudinal ridges; disc more or less weakly impressed; suture elevated in posterior three-fourths; macropunctation coarse, irregular, partly confluent, and partly somewhat seriate; interstices with shallow and irregular micropunctation. Hind wings fully developed. Protarsomeres I–IV moderately dilated.

Abdomen with fine and dense punctation on tergites III–VI; tergite VIII with dense pubescence, posterior margin of tergite VIII broadly and weakly convex (Fig. 6C); interstices with distinct microreticulation; posterior margin of tergite VII with palisade fringe.

Male. Sternites III–VI unmodified; sternite VII (Fig. 6F) distinctly transverse, with very shallow median impression posteriorly, this impression with sparse modified black setae, posterior margin broadly concave; sternite VIII (Fig. 6G) with shallow median impression posteriorly, this impression with stout black setae, posterior excision deep, almost V-shaped, on either side of the posterior excision with dense cluster of dark setae; aedeagus as in Figs 6H–J, ventral process evenly curved and apically acute in lateral view; dorsal fig long, apical portion distinctly sclerotized and apically acute in lateral view, basal portion short.

Female. Posterior margin of sternite VIII (Fig. 6D) broadly convex; genital segments with an asymmetric and weakly sclerotized structure (Fig. 6E).

#### Comparative notes.

The similar chaetotaxy and shape of the male sternite VIII and the similar shape of the ventral process of the aedeagus suggest that *Domene malaisei* is closely allied to *Domene reducta*. *Domene malaisei* is readily distinguished from other species of the group by on average darker coloration, smaller body size, the deeper posterior excision of the male sternite VIII, the evenly curved ventral process of the aedeagus and by the shape of the sclerotized structure in the female genital segments.

#### Distribution and natural history.

The currently known distribution is confined to the type locality Kambaiti in northeastern Myanmar at the border with Yunnan, and two localities in western Yunnan (Fig. 1). The examined non-type specimens were sifted from forest leaf litter at altitudes of 2000–2500 m.

### Domene (Macromene) reitteri

Taxon classificationAnimaliaColeopteraStaphylinidae

Koch, 1939

Figs 1 , 7A , 8 , 9

Domene (Macromene) reitteri Koch, 1939: 161

#### Type material examined.

Lectotype ♂, present designation: “Tienmuschan, N. W China, Rtt. / Type / Domene reitteri Koch det. C. Koch / Holotype 1956 det. Kamp / Holotypus Domene reitteri Koch / Domene reitteri Koch V. L. Gusarov det. 1993 / Lectotypus ♂, *Domene reitteri* Koch, desig. B. Feldmann 2010” (NHMB).

Paralectotypes 3 ♀♀: „Tienmuschan, N. W China, Rtt. / Cotype / Paratypus Domene reitteri Koch / Domene reitteri Koch V. L. Gusarov det. 1993“; 1 ex. (abdomen missing): “Tienmuschan, N. W China, Rtt. / Cotype” (NHMB).

#### Comment.

The original description of *Domene reitteri* is based on an unspecified number of syntypes from “Tienmuschan (nordwestliches [sic] China) ex coll. E. Reitter” (Koch 1939). Five syntypes, one male, three females and one unsexed specimen, were located in the Koch collection at the Naturhistorisches Museum Basel. The male syntype is designated as the lectotype.

#### Additional material examined

(87 ♂♂, 59 ♀♀)**. China: Zhejiang:** 11 ♂♂, 7 ♀♀, Anji County, Longwang Shan, 30°23'59"N 119°26'26"E, 1300–1450 m, 14.V.2013, leg. Hu (SNUC); 31 ♂♂, 14 ♀♀, Longwang Shan, 30°24'28"N 119°26'37"E, 1050–1200 m, 15.V.2013, leg. Li & al. (SNUC); 15 ♂♂, 7 ♀♀, Longwang Shan, Qianmutian, 30°24'N 119°26'E, 1050–1250 m, 08.VI.2012, leg. Yin & Hu (SNUC, cAss); 1 ♂, Longwang Shan, 30°24'N 119°26'E, 1250–1450 m, 14.V.2013, leg. Chen & Pan (SNUC); 3 ♂♂, 5 ♀♀, Longwang Shan, Dongguan, 1250 m, 26.V.2009, leg. Feng & al. (SNUC); 3 ♂♂, 6 ♀♀, Longwang Shan, Qianmutian, 1300 m, 24.V.2009, leg. Feng & al.(SNUC); 8 ♂♂, 10 ♀♀, Longwang Shan, 950–1200 m, 25.IV.2006, leg. He (SNUC); 2 ♂♂, 3 ♀♀, Longwang Shan, Qianmutian, 1300 m, 29.V.2009, leg. Feng & al.(SNUC); 1 ♀, Longwang Shan, Qianmutian, 700–1325 m, 28.VII.2011, leg. Pan (SNUC); 1 ♂, 1 ♀, Tianmu Shan, 1200–1300 m, 25.–29.VII.2011, leg. Chen (SNUC); 2 ♂♂, 2 ♀♀, Tianmu Shan, 300 m, 17.V.2006, leg. Hu & Tang (SNUC); 1 ♀, Tianmu Shan, 1100 m, 24.VII.2011, leg. Hu & Tang (SNUC); 1 ♂, Tianmu Shan, 1500 m, 15.VIII.2010, leg. Hu (SNUC); 1 ♂, Tianmu Shan, 300–400 m, 29.V.2010, leg. Wang (SNUC); 1 ♂, East Tianmu Shan, 1050–1150 m, 13. IV.2011, leg. Peng & Zhu (SNUC); 2 ♂♂, Tianmu Shan, 13.VI.2009, leg. Song (SNUC); 2 ♂♂, Tianmu Shan, 1000 m, 2.V.2009, leg. Song (SNUC); 2 ♂♂, 1 ♀, Tianmu Shan, 1500 m, 15.VIII.2010, leg. Hu (SNUC); 2 ♂♂, 1 ♀, West Tianmu Shan N.R., path to peak of immortals (“Blind Alley”), 30°20'34"N, 119°25'51"E, 1100–1200 m, primary mixed forest, litter moss, sifted, 15.VI.2007, leg. Wrase (cSch, cFel).

#### Redescription.

Measurements (in mm) and ratios: TL 5.78–8.62, FL 4.16–4.43, HL 1.07–1.17, HW 1.05–1.11, AnL 2.78–3.05, NW 0.37–0.41, PL 1.28–1.35, PW 1.02–1.06, EL 1.07–1.13, EW 1.12–1.20, TiL 1.39–1.44, TaL 0.89–0.96, AW 1.12–1.24, AL 1.07–1.17, HL/HW 1.02–1.05, HW/PW 1.03–1.05, HL/PL 0.84–0.87, NW/HW 0.35–0.37, PL/PW 1.25–1.28, EL/PL 0.84–0.89.

**Figure 7.**
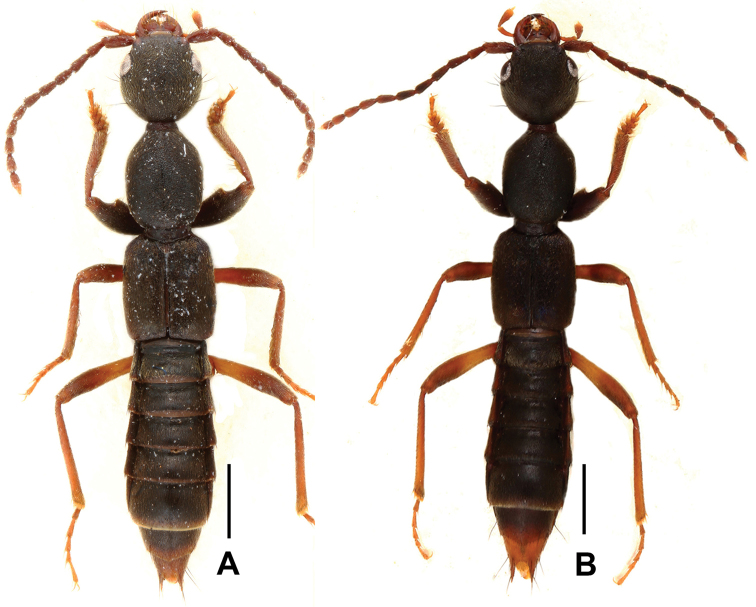
Habitus of *Domene* spp., **A** *Domene reitteri* **B** *Domene chenae*. Scales: 1.0 mm.

**Figure 8.**
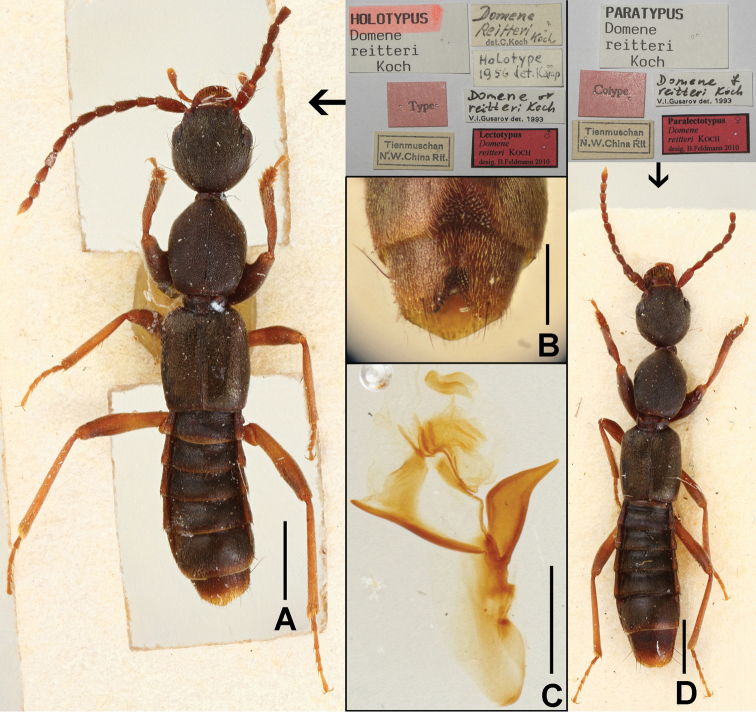
*Domene reitteri*. **A** lectotype **B** male sternite VII–VIII **C** aedeagus of lectotype in lateral view **D** paralectotype. Scales: **A, D** 1.0 mm; **B**–**C** 0.5 mm.

Habitus as in Figs 7A, 8A, 8D. Body blackish brown; legs with dark brown profemora and protibiae, basal halves of metafemora light brown, distal halves gradually infuscate; antennae brown to light brown.

**Figure 9.**
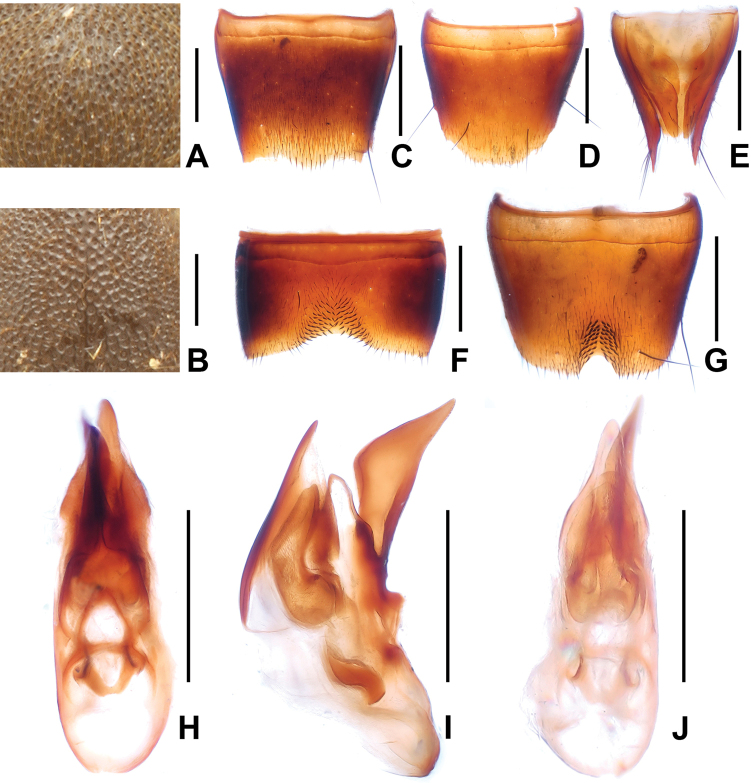
*Domene reitteri*. **A** median dorsal portion of head **B** median portion of pronotum **C** female tergite VIII **D** female sternite VIII **E** female tergites IX–X. **F** male sternite VII **G** male sternite VIII **H** aedeagus in ventral view **I** aedeagus in lateral view **J** aedeagus in dorsal view. Scales: **A**–**B** 0.2 mm; **C**–**J** 0.5 mm.

Head orbicular, broadest across eyes; punctation (Fig. 9A) moderately coarse, weakly umbilicate, and very dense, interstices forming very narrow ridges. All antennomeres longer than broad; antennomeres IV–X of equal length; antennomere I 1.6 times, II 1.1 times, III 1.3 times, XI 1.3 times as long as IV. Maxillary palpus very slender, preapical joint 2.7–3.2 times as long as broad.

Pronotum slightly narrower than head, widest in the middle; lateral margins convex in dorsal view; punctation (Fig. 9B) somewhat coarser than that of head; midline with rudiment of a fine glossy line.

Elytra without distinct longitudinal ridges; suture weakly elevated; punctation very fine, dense and uniform; hind wings reduced. Protarsomeres I–IV dilated in both sexes.

Abdomen with punctation fine and dense on tergites III–VI, finer and somewhat sparser on tergite VIII, posterior margin of tergite VIII weakly convex in the middle (Fig. 9C); interstices with shallow microreticulation; posterior margin of tergite VII with palisade fringe.

Male. Sternites III–VI unmodified; sternite VII (Figs 8B, 9F) distinctly transverse, with median impression of triangular shape posteriorly, this impression with numerous distinctly modified, short and stout black setae; posterior margin distinctly concave in the middle; sternite VIII (Figs 8B, 9G) transverse, with pronounced and symmetric impression posteriorly, this impression with distinctly modified short and stout black setae, posterior excision small and U-shaped; aedeagus as in Figs 8C, 9H–J, ventral process stout and apically acute; dorsal fig with large and lamellate apical portion, and with short, thin basal portion; internal sac with membranous structures.

Female. Posterior margin of sternite VIII (Fig. 9D) broadly convex; genital segments with weakly asymmetric large and moderately sclerotized structure (Fig. 9E).

#### Comparative notes.

The fine, dense and uniform punctation of the elytra, and the similar shape and chaetotaxy of the male sternite VII and sternite VIII suggest that *Domene reitteri* is most closely allied to *Domene chenae*. It is distinguished from *Domene chenae* by the finer punctation of the head and pronotum, the numerous distinctly modified, short and stout black setae on the male sternite VII, the stouter ventral process of the aedeagus and by the shape of the sclerotized structure in the female genital segments.

#### Distribution and natural history.

The distribution is confined to several localities in the Tianmu Shan range in the northwest of Zhejiang. The specimens were sifted from leaf litter in broad-leaved and primary mixed forests at altitudes of 300–1500 m.

### Domene (Macromene) chenae

Taxon classificationAnimaliaColeopteraStaphylinidae

Peng & Li sp. n.

http://zoobank.org/6F65882A-3C00-4988-A3C6-6FC9B0D57C9B

Figs 1 , 7B , 10

#### Type material

(2 ♂♂, 1 ♀). Holotype ♂: “China: Guangxi Prov., Lingui County, Huping N. R., Anjiangping, 25°34'N, 109°57'E, 13.VII.2011 1,200 m, Zhu, Chen & Ma leg. / Holotypus ♂ *Domene chenae* sp. n., det Peng & Li. 2014” (SNUC). Paratypes: 1 ♂: same data as holotype (SNUC); 1 ♀: same data, but “He & Tang leg.” (SNUC).

#### Etymology.

The species is named after Yan Chen, who collected some of the type specimens.

#### Description.

Measurements (in mm) and ratios: BL 7.95–8.17, FL 4.55–4.73, HL 1.20–1.24, HW 1.14–1.17, AnL 3.17–3.39, NW 0.43–0.46, PL 1.30–1.37, PW 1.07–1.09, EL 1.11–1.13, EW 1.22–1.24, TiL 1.58–1.66, TaL 0.94–1.02, AW 1.26–1.30, AL 1.12, HL/HW 1.05–1.06, HW/PW 1.06–1.07, HL/PL 0.91–0.92, NW/HW 0.38–0.39, PL/PW 1.21–1.26, EL/PL 0.82–0.85.

Habitus as in Fig. 7B. Body black with distinctly paler abdominal apex; legs with blackish brown profemora and dark brown protibiae, basal halves of metafemora light brown, distal halves gradually infuscate; antennae brown to light brown.

**Figure 10.**
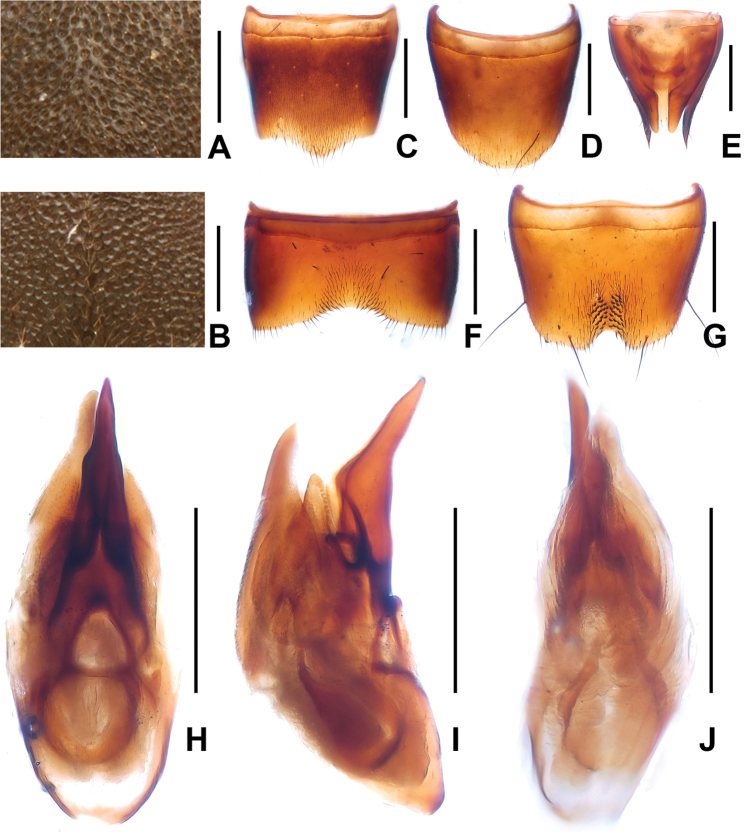
*Domene chenae*. **A** median dorsal portion of head **B** median portion of pronotum **C** female tergite VIII **D** female sternite VIII **E** female tergites IX–X. **F** male sternite VII **G** male sternite VIII **H** aedeagus in ventral view **I** aedeagus in lateral view **J** aedeagus in dorsal view. Scales: **A**–**B** 0.2 mm; **C**–**J** 0.5 mm.

Head orbicular, widest across eyes; punctation (Fig. 10A) coarse, distinctly umbilicate, and very dense, interstices forming very narrow ridges. All antennomeres longer than broad; antennomeres IV–X of equal length; antennomeres I 1.6 times, II 0.9 times, III 1.1 times, XI 1.2 times as long as IV. Maxillary palpus very slender, preapical joint 2.8–2.9 times as long as broad.

Pronotum narrower than head, widest in the middle; lateral margins weakly convex in dorsal view; punctation (Fig. 10B) similar to that of head; midline with rudiment of a fine glossy line.

Elytra without distinct longitudinal ridges; suture elevated in posterior two thirds; punctation fine, dense and uniform; interstices without micropunctation. Hind wings probably present. Protarsomeres I–IV moderately dilated.

Abdomen with fine and dense punctation on tergites III–VIII; posterior margin of tergite VIII broadly and weakly convex (Fig. 10C); interstices with shallow microreticulation; posterior margin of tergite VII with palisade fringe.

Male. Sternites III–VI unmodified; sternite VII (Fig. 10F) distinctly transverse, with median impression of triangular shape posteriorly, this impression with moderately modified dark setae, posterior margin broadly concave in the middle; sternite VIII (Fig. 10G) transverse, with shallow median impression posteriorly, this impression with distinctly modified stout and black setae, posterior excision small and U-shaped; aedeagus as in Figs 10H–J, ventral process more slender and curved, apically acute; dorsal fig with distinctly sclerotized apical portion, basal portion short.

Female. Posterior margin of sternite VIII (Fig. 10D) broadly convex; genital segments with weakly asymmetric, large and sclerotized structure (Fig. 10E).

#### Comparative notes.

The fine, dense and uniform punctation of the elytra, and the similar shape and chaetotaxy of the male sternite VII and sternite VIII suggest that *Domene chenae* is allied to *Domene reitteri*. The species is distinguished from *Domene reitteri* by the coarser punctation of the head and pronotum, the somewhat shorter elytra, the moderately modified dark setae of the male sternite VII, the differently shaped ventral process of the aedeagus and the more distinctly sclerotized structure in the female genital segments.

#### Distribution and natural history.

The type locality is situated in Anjiangping to the northwest of Guilin, northern Guangxi (Fig. 1). The specimens were sifted from leaf litter and grass in broad-leaved forests at an altitude of 1200 m.

### Domene (Macromene) cultrata

Taxon classificationAnimaliaColeopteraStaphylinidae

Feldmann & Peng sp. n.

http://zoobank.org/4792CF08-CAC7-44BF-9293-060F1E1DBA61

Figs 1 , 11A , 12

#### Type material

(10 ♂♂, 12 ♀♀). Holotype: ♂, “China (Shaanxi) Qin Ling Shan, 110.06 E, 34.27 N, Hua Shan, 118 km E Xian, N valley, 1200–1400 m, leafy wd.sifted, 18./20.VIII.1995, Wrase / Sammlung M. Schülke Berlin / Holotypus ♂ *Domene cultrata* sp. n., det. B. Feldmann & Z. Peng 2014 ” (cSch). Paratypes: 2 ♂♂, 3 ♀♀ (4 specimens are teneral): same label data as holotype (cSch, cRou, cFel);; 1 ♂: “China [28] S-Shaanxi, 34 km S Hanzhong, 32°44'22"N, 106°51'55"E, 1460 m, 14.VIII.2012, V. Assing” (cAss); 1 ♂: “China [27a] S-Shaanxi, Micang Shan, 42 km S Hanzhong, 32°40'52"N, 106°49'16"E, 1090 m, 14.VIII.2012, V. Assing” (cFel); 2 ♀♀: “China (S. Shaanxi), Micang Shan, 42 km S Hanzhong, 32°40'43"N, 106°48'33"E, 1090 m, (stream valley, shady S. slope, sec. mixed for., raked from roots of perennials, soil, under stones) 17.VIII.2012, D.W. Wrase (32)” (cSch, cFel); 1 ♂, 1 ♀: “China: Shaanxi, Qin Ling Shan, 110.06 E, 34.27 N, Hua Shan Mt. N Valley, 1200–1400 m, 118 km E Xian, sifted, 18./20.VIII.1995, leg. M. Schülke” (cSch); 1 ♀: “China: border Shaanxi–Sichuan [today Chongqing], Daba Shan pass, 20 km SSE Zhenping, 1700–1800 m, 31°44'N, 109°35'E, 9.VII.2001, A. Smetana [C96b]” (cSme); 1 ♂, 1 ♀: “China: Shaanxi Prov., Zhouzhi County, Houzhenzi, Qinling, West Sangongli Gou, N33.50.613 E107.48.524 / 17–19.V.2008 alt. 1,336 m, Hao Huang & Xu Wang leg.” (SNUC); 1 ♂, 2 ♀♀: “China [17] S-Gansu, S Longnan, Min Shan, macchia, 33°05'24"N, 104°45'13"E, 1500 m, 6.VIII.2012, V. Assing”(cAss); 2 ♂♂, 2 ♀♀: “China (W-Hubei) Daba Shan, creek vall. 8 km NW Muyuping, 31°29'N, 110°22'E, 1540 m, (edge of small creek), 18.VII.2001, Wrase (16)” (cSch, cFel).

#### Etymology.

The specific epithet is an adjective derived from the Latin noun culter (knife) and alludes to the shape of the ventral process of the aedeagus.

#### Description.

Measurements (in mm) and ratios: BL 8.90–10.2, FL 5.38–5.50, HL 1.31–1.50, HW 1.22–1.39, AnL 3.22–3.62, NW 0.46–0.50, PL 1.48–1.62, PW 1.17–1.40, EL 1.46–1.63, EW 1.50–1.78, TiL 1.65–1.92, TaL 1.18–1.42, AW 1.37–1.53, AL 1.33–1.48, HL/HW 1.04–1.14, HW/PW 0.99–1.07, HL/PL 0.88–0.96, NW/HW 0.36–0.38, PL/PW 1.19–1.26, EL/PL 0.99–1.01.

**Figure 11.**
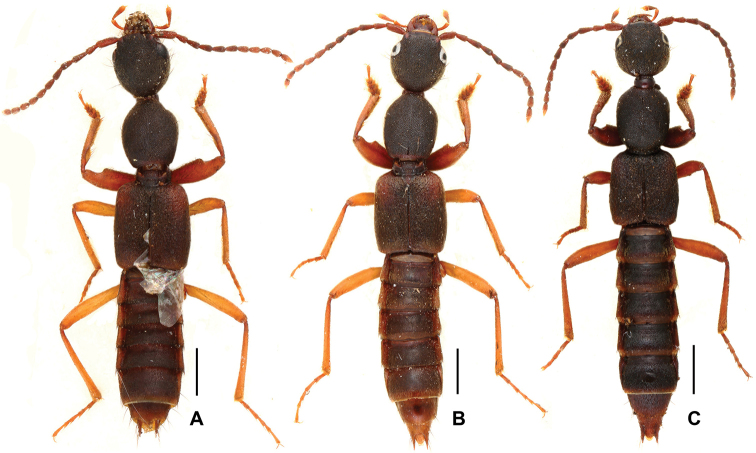
Habitus of *Domene* spp., **A** *Domene cultrata* **B** *Domene cuspidata* **C** *Domene reducta*. Scales: 1.0 mm.

Habitus as in Fig. 11A. Body dark brown; legs brownish yellow, with brown profemora and protibiae; antennae brown to light brown.

**Figure 12.**
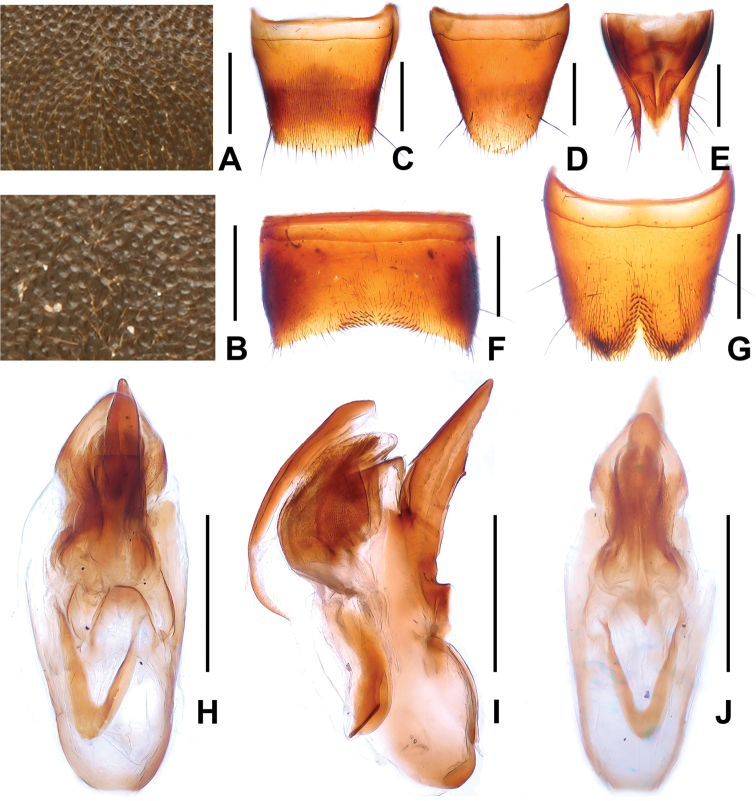
*Domene cultrata*. **A** median dorsal portion of head **B** median portion of pronotum **C** female tergite VIII **D** female sternite VIII **E** female tergites IX–X. **F** male sternite VII **G** male sternite VIII **H** aedeagus in ventral view **I** aedeagus in lateral view **J** aedeagus in dorsal view. Scales: **A**–**B** 0.2 mm; **C**–**J** 0.5 mm.

Head orbicular, widest behind eyes; punctation (Fig. 12A) coarse, umbilicate and dense, interstices forming very narrow ridges. All antennomeres longer than broad; antennomeres IV–X of equal length; antennomeres I 1.6 times, II 0.9 times, III 1.3 times, XI 1.4 times as long as IV. Maxillary palpus very slender, preapical joint 2.8–3.0 times as long as broad.

Pronotum about as wide as head, widest in the middle; lateral margins convex in dorsal view; punctation (Fig. 12B) similar to that of head; midline with rudiment of a fine glossy line.

Elytra without distinct longitudinal ridges; suture elevated in posterior two thirds; macropunctation coarse, irregular, partly confluent, and partly somewhat seriate; interstices rugose, rendering elytra matt, with irregular and mostly barely visible micropunctation (visible in posterior part of elytra). Hind wings fully developed. Protarsomeres I–IV distinctly dilated.

Abdomen with fine and dense punctation on tergites III–VIII; posterior margin of tergite VIII broadly convex (Fig. 12C); interstices with distinct microreticulation; posterior margin of tergite VII with palisade fringe.

Male. Sternites III–VI unmodified; sternite VII (Fig. 12F) distinctly transverse, with shallow median impression posteriorly, this impression with sparse, strongly modified, short and stout black setae, posterior margin broadly concave; sternite VIII (Fig. 12G) with shallow median impression, this impression with distinctly modified stout black setae, posterior excision moderately deep and V-shaped, on either side of the posterior excision with dense cluster of dark setae; aedeagus as in Figs 12H–J, ventral process nearly straight and apically acute; dorsal fig with long, large and distinctly sclerotized apical portion, basal portion short and lamellate; internal sac with small sclerotized spines and with distinct membranous structures.

Female. Sternite VIII (Fig. 12D) distinctly oblong, posterior margin strongly convex; genital segments with asymmetric, slender and moderately sclerotized structure (Fig. 12E).

#### Intraspecific variation.

*Domene cultrata* is subject to rather pronounced intraspecific variation of size, body proportions and coloration of the legs.

#### Comparative notes.

Based on the similar chaetotaxy and shape of the male sternite VIII, and the shape of the ventral process of the aedeagus, *Domene cultrata* belongs to the *Domene malaisei* species group and is allied to *Domene cuspidata*. It can be distinguished from other species of the group by the distinctly coarser macropunctation of the elytra, the differently shaped ventral process of the aedeagus, and the slender sclerotized structure in the female genital segments, from *Domene malaisei* and *Domene reducta* also by the shallower impression and the less deep posterior excision of the male sternite VIII.

#### Distribution and natural history.

This species has been recorded from the Qinling Shan and Daba Shan, as well as from adjacent mountain ranges (Fig. 1). The specimens were sifted from leaf litter in forests or raked from roots of perennials and soil, or found under stones at altitudes of 1090–1800 m. Four specimens found in August are teneral.

### Domene (Macromene) cuspidata

Taxon classificationAnimaliaColeopteraStaphylinidae

Feldmann & Peng sp. n.

http://zoobank.org/6E9BB0EB-775C-4C0B-BDB4-8F2B43FDEF26

Figs 1 , 11B , 13

#### Type material

(10 ♂♂, 24 ♀♀). Holotype: ♂: “China: Shaanxi Prov., Hanzhong City, Nanzheng County, Yuanba Town, Liping National Forest Park / 32°50'N, 106°36'E, 15.VII.2012 1,400–1,600 m, Chen, Li, Ma, & Zhao leg. / Holotypus ♂ *Domene cuspidata* sp. n., det. B. Feldmann & Z. Peng 2014” (SNUC). Paratypes: 2 ♂♂, 11 ♀♀: same data as holotype (SNUC, cAss); 1 ♀: same data, but “16.VII.2012, Yu-Hong Pan leg.” (SNUC); 1 ♀: same data, but “16.VII.2012, Li-Zhen Li leg.” (SNUC); 3 ♀♀: same data, but “16.VII.2012” (SNUC); 1 ♀: “China, Shaanxi, Qinling Shan above Houzhenzi, 115 km WSW Xi’an, 1450 m, 33°50'N, 107°47'E, 5.VII.2001, A. Smetana [C95b]” (cSme); 1 ♂: “China [3] S-Gansu, N Chengxian, W-Qinling Shan, 34°08'24"N, 105°46'43"E, 1750 m, 28.VII.2012, V. Assing” (cAss); 1 ♂: “China: S-Gansu [CH 12-03], W Qinling Shan, 43 km N Chengxian, 34°08'24"N, 105°46'43"E, 1750 m, moist valley with creek and ponds, meadow with Artemisia, 28.VII.2012, leg M. Schülke” (cSch); 1 ♀: “China: S-Gansu [CH 12-05], W Qinling Shan, 47 km N Chengxian, 34°10'17"N, 105°42'56"E, 1850 m, mixed secondary forest margin, litter sifted, 29.VII.2012, leg M. Schülke” (cSch); 1 ♂, 1 ♀: “China, S-Gansu [CH 12-05], W. Qinling Shan, 47 km N Chengxian, 34°10'20"N, 105°42'19"E, 1830 m, (creek valley, loam deposit on meadow with tall herbaceous vegetation,raked/dug, 29.VII.2012, D. W. Wrase” (cSch, cFel); 1 ♂, 3 ♀♀: “China: W-Sichuan, Ya’an Pref., Shimian Co., Daxue Shan, road betw. Anshunchang–Wanba, 12 km W Shimian, 1300 m, 9.VII.1999, leg. A. Pütz” (cPüt, cFel).

#### Etymology.

The specific epithet is an adjective derived from the Latin noun cuspis (cusp) and refers to the apically acute ventral process of the aedeagus.

#### Description.

Measurements (in mm) and ratios: BL 8.89–9.56, FL 5.12–5.34, HL 1.26–1.39, HW 1.20–1.30, AnL 3.17–3.61, NW 0.45–0.50, PL 1.48–1.57, PW 1.20–1.28, EL 1.42–1.50, EW 1.48–1.62, TiL 1.72–1.79, TaL 1.20–1.33, AW 1.35–1.49, AL 1.32–1.65, HL/HW 1.04–1.08, HW/PW 1.00–1.03, HL/PL 0.85–0.89, NW/HW 0.38–0.39, PL/PW 1.21–1.24, EL/PL 0.94–0.97.

**Figure 13.**
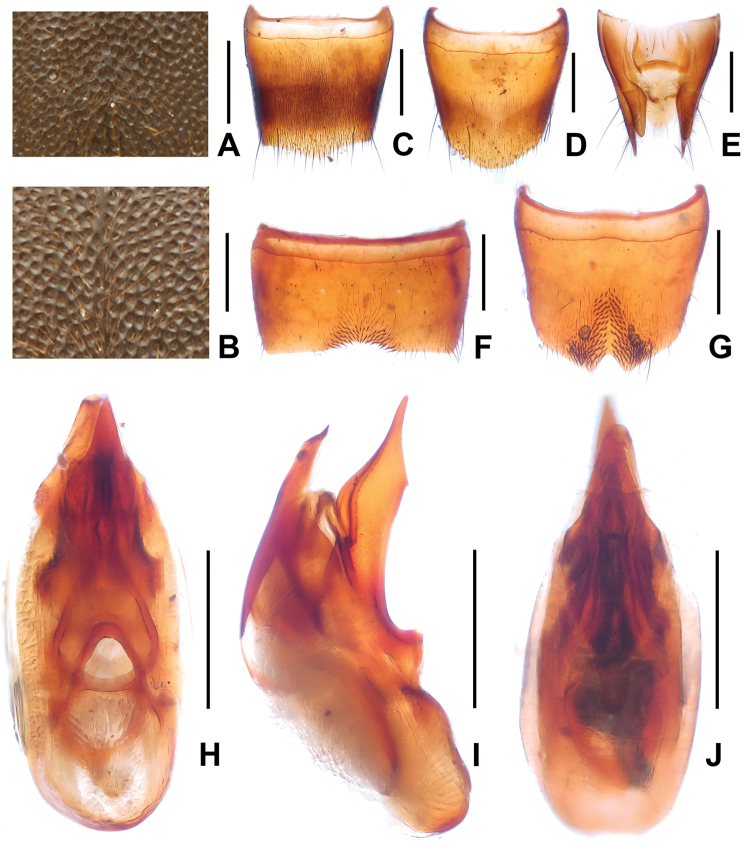
*Domene cuspidata*. **A** median dorsal portion of head **B** median portion of pronotum **C** female tergite VIII **D** female sternite VIII **E** female tergites IX–X. **F** male sternite VII **G** male sternite VIII **H** aedeagus in ventral view **I** aedeagus in lateral view **J** aedeagus in dorsal view. Scales: **A**–**B** 0.2 mm; **C**–**J** 0.5 mm.

External characters (Fig. 11B) as in *Domene cultrata*, distinguished only by the distinctly less coarse macropunctation and less rugose interstices of the elytra rendering the elytra more shiny in *Domene cuspidata*, and by the primary and secondary sexual characters:

Male. Sternites III–VI unmodified; sternite VII (Fig. 13F) distinctly transverse, with shallow postero-median impression, this impression with sparse strongly modified, short and stout black setae, posterior margin concave in the middle; sternite VIII (Fig. 13G) with extensive median impression, this impression with distinctly modified stout black setae, posterior excision less deep, V-shaped, on either side of the posterior excision with a dense cluster of dark setae; aedeagus as in Figs 13H–J, ventral process distinctly sclerotized, with slender and very acute apical portion; dorsal fig with long, large and distinctly sclerotized apical portion, basal portion short; internal sac with membranous structures.

Female. Sternite VIII (Fig. 13D) oblong, posterior margin broadly convex; genital segments with asymmetric, large and moderately sclerotized structure (Fig. 13E).

#### Comparative notes.

Based particularly on the similar chaetotaxy and shape of the male sternite VIII and the shape of the ventral process of the aedeagus, *Domene cuspidata* belongs to the *Domene malaisei* species group and is closely allied to *Domene cultrata*. It is distinguished from other species of the group by the apically more acute ventral process of the aedeagus and by the large, moderately sclerotized structure in the female genital segments, from *Domene malaisei* and *Domene reducta* also by the shallower impression and less deep posterior excision of the male sternite VIII.

#### Distribution and natural history.

The species was recorded from the Qinling Shan and Dalou Shan (Fig. 1). The specimens were sifted from forest leaf litter and a loamy meadow with tall herbaceous vegetation at altitudes of 1300–1850 m. Six paratypes found in July are teneral.

### Domene (Macromene) reducta

Taxon classificationAnimaliaColeopteraStaphylinidae

Feldmann & Peng sp. n.

http://zoobank.org/17A30E81-7F93-4329-AF1B-32CE17E380E9

Figs 1 , 11C , 14

#### Type material

(5 ♂♂, 9 ♀♀). Holotype ♂: “China: Sichuan Prov., Tianquan County, Labahe N. R., 30°09'N, 102°27'E, 29.VII.2006 1,900 m, Hu & Tang leg. / Holotypus ♂ *Domene reducta* sp. n., det. B. Feldmann & Z. Peng 2014” (SNUC). Paratypes: 1 ♂, 5 ♀♀ [all teneral], same label data as holotype (SNUC); 2 ♂♂, 1♀ [1 ♂, 1 ♀ teneral]: same data, but “Liangluxiang, 29°56'N, 102°23'E, alt. 1,500–1,700 m / 10.VII.2012, Dai, Peng & Yin leg.” (SNUC); 1 ♀ [teneral]: same data, but “Liangluxiang, 29°56'N, 102°23'E, alt. 1,900–2,000 m, 10.VII.2012, Dai, Peng & Yin leg leg.” (SNUC); 1 ♂: “China, Sichuan: Quing-cheng-Shan [ca. 30°53'N, 103°35'E], 1400–1700 m, 22.VI.1996, D. Erber” (cFel); 1 ♀: “China, W.Sichuan, (Ya’an Pref. Tianquan Co.), Jiajin Shan, valley above Labahe, N.R.ST., 57 km W Ya’an, 30°06'N, 102°25'E (light forest), 1800 m, 12.VII.1999, D.W. Wrase” (cFel); 1 ♀: “China: W-Sichuan, Ya’an Prefecture, Tianquan Co., Jiajin Shan, Tal oberh. Labahe, N.R.St., 57 km W. Ya’an, 30°06'N 102°25'E, Streu, Rinde, Pilze, 1800 m, 12.VII.1999, leg. M. Schülke” (cSch).

#### Etymology.

The specific epithet (Latin, adjective: reduced) alludes to the minute sclerotized structure in the female genital segments.

#### Description.

Measurements (in mm) and ratios: BL 8.95–10.84, FL 5.37–5.48, HL 1.42–1.48, HW 1.35–1.41, AnL 3.36–3.56, NW 0.50–0.55, PL 1.57–1.66, PW 1.28–1.35, EL 1.41–1.48, EW 1.63–1.70, TiL 1.81–1.87, TaL 1.28–1.31, AW 1.51–1.57, AL 1.52–1.54, HL/HW 1.04–1.06, HW/PW 1.03–1.05, HL/PL 0.89–0.91, NW/HW 0.37–0.39, PL/PW 1.22–1.24, EL/PL 0.88–0.91.

Habitus as in Fig. 11C. Body dark brown; legs light brown with darker profemora and protibiae; antennae brown to light brown.

**Figure 14.**
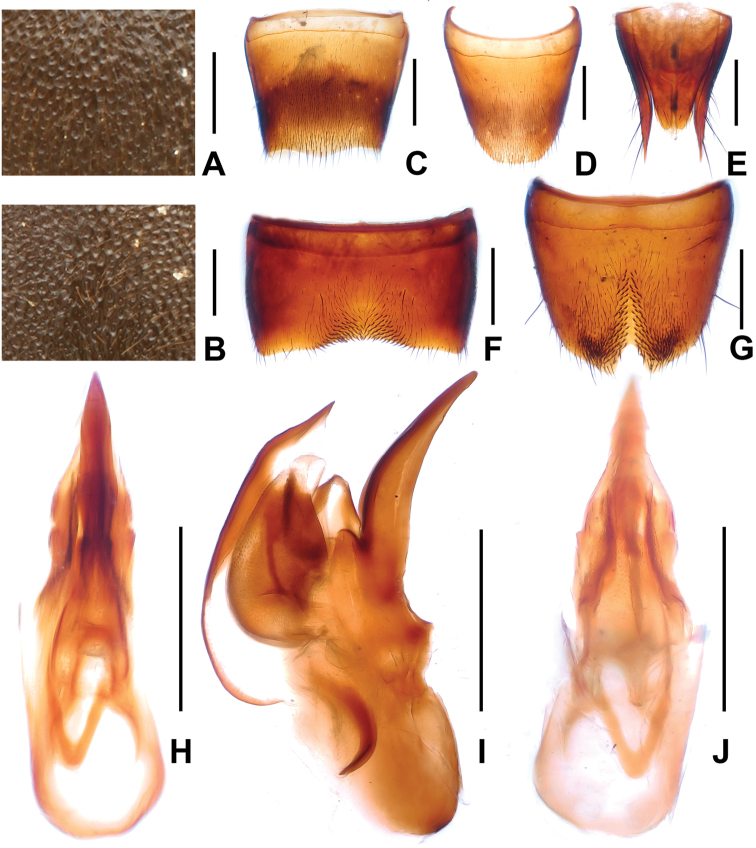
*Domene reducta*. **A** median dorsal portion of head **B** median portion of pronotum **C** female tergite VIII **D** female sternite VIII **E** female tergites IX–X. **F** male sternite VII **G** male sternite VIII **H** aedeagus in ventral view **I** aedeagus in lateral view **J** aedeagus in dorsal view. Scales: **A**–**B** 0.2 mm; **C**–**J** 0.5 mm.

Head orbicular, widest behind eyes; punctation (Fig. 14A) moderately coarse, distinctly umbilicate, and very dense, interstices forming very narrow ridges. All antennomeres longer than wide; antennomeres IV–X of equal length; antennomeres I 1.7 times, II 1.1 times, III 1.4 times, XI 1.2 times as long as IV. Maxillary palpus slender, preapical joint 3.2–3.5 times as long as broad.

Pronotum slightly narrower than head, widest in the middle; lateral margins convex in dorsal view; punctation (Fig. 14B) somewhat coarser than that of head; midline with rudiment of a fine glossy line.

Elytra without distinct longitudinal ridges; suture elevated in posterior three-fourths; macropunctation moderately coarse, irregular, partly confluent, and partly somewhat seriate; interstices with irregular micropunctation. Hind wings fully developed. Protarsomeres I–IV distinctly dilated.

Abdomen with fine and dense punctation on tergites III–VIII; posterior margin of tergite VIII broadly and weakly convex (Fig. 14C); interstices with shallow microreticulation; posterior margin of tergite VII with palisade fringe.

Male. Sternites III–VI unmodified; sternite VII (Fig. 14F) distinctly transverse, with median impression of triangular shape posteriorly, this impression with strongly modified, short and stout black setae, posterior margin weakly concave in the middle; sternite VIII (Fig. 14G) with shallow and extensive median impression, this impression with stout black setae, posterior excision moderately deep and V-shaped, on either side of the posterior excision with a dense cluster of dark setae; aedeagus as in Figs 14H–J, ventral process long, slender, evenly curved and apically acute; dorsal fig with long and distinctly sclerotized apical portion, basal portion long and lamellate.

Female. Posterior margin of sternite VIII (Fig. 14D) broadly convex; genital segments with a small symmetric, weakly sclerotized structure (Fig. 14E).

#### Comparative notes.

Based particularly on the similar chaetotaxy and shape of the male sternite VIII, and the shape of the ventral process of the aedeagus, *Domene reducta* belongs to the *Domene malaisei* species group and is closely related to *Domene malaisei*. *Domene reducta* is distinguished from other species of the group by on average larger body size (especially from *Domene malaisei*), the shape of the impression on the male sternite VIII, the long, slender, evenly curved ventral process of the aedeagus and by the symmetric, small and moderately sclerotized structure in the female genital segments.

#### Distribution and natural history.

The species is known from the Qingcheng Shan and Hengduan Shan, central Sichuan (Fig. 1). The specimens were sifted from leaf litter and soil in evergreen broad-leaved forests at altitudes of 1400–1900 m. Nine paratypes found in July are teneral.

### Domene (Macromene) sp.

Taxon classificationAnimaliaColeopteraStaphylinidae

#### Material studied.

5♀♀, Sichuan, Emei Shan, 29°34'N, 103°21'E, 1800–2400 m, sifted, 27.VI.–5.VII.2009, leg. Grebennikov (cSme, cAss).

#### Comment.

The above brachypterous females undoubtedly represent an undescribed species distinguished from the other species known from China by the conspicuously large head and the distinctly impressed sutural portion of the elytra, from most species also by the short and narrow elytra and by the absence of a palisade fringe at the posterior margin of the male tergite VII.

### Key to the *Domene* species of China

Because of some variability in size, body proportions, coloration, punctation and sculpture in most species, a positive identification (especially of the species of the *malaisei* group) requires the examination of the genitalia.

**Table:** 

1	Head of flattened, subcircular shape. Male sternite VIII (Fig. 4G) with pronounced median impression with numerous distinctly modified short and stout black setae; aedeagus (Figs 4H–J) large (1.63–1.65 mm) with completely reduced dorsal fig. Posterior margin of female sternite VIII (Fig. 4D) with distinct median concavity. China: western Zhejiang (Fig. 1)	***Domene firmicornis* Assing & Feldmann, 2014**
–	Head less strongly flattened and of orbicular shape. Chaetotaxy and shape of male sternite VIII different; aedeagus smaller (< 1.60 mm) and with distinct dorsal fig. Female sternite VIII with more or less convex posterior margin	**2**
2	Smaller species; length of forebody ≤ 4.73 mm. Punctation of head and especially pronotum fine; male sternite VIII with small to very small (*Domene chenpengi*) U-shaped excision posteriorly	**3**
–	Larger species; length of forebody ≥ 4.70 mm. Punctation of head and pronotum coarser. Male sternite VIII of different shape and chaetotaxy	**5**
3	Punctation of pronotum very fine with interstices forming narrow, longitudinal ridges. Male sternite VII (Fig. 3F) with only few distinctly modified setae; male sternite VIII (Fig. 3G) with very small posterior excision; ventral process of aedeagus (Figs 3H–J) with relatively short and less stout apical portion. Female genital segments (Fig. 3E) with relatively small sclerotized structure. Russian Far East; South Korea; China: Beijing, Jilin (Fig. 1)	***Domene chenpengi* Li, 1990**
–	Punctation of pronotum coarser. Male sternite VII with more numerous modified setae; male sternite VIII with deeper U-shaped excision. Female genital segments with more pronounced sclerotized structure	**4**
4	Punctation of head and pronotum (Figs 10A–B) coarser. Male sternite VII (Fig. 10F) with numerous moderately modified dark setae; ventral process of aedeagus (Figs 10H–J) with slender apical portion (in lateral view). Female genital segments (Fig. 10E). China: Guangxi (Fig. 1)	***Domene chenae* sp. n.**
–	Punctation of head and pronotum less coarse (Figs 9A–B). Male sternite VII (Figs 8B, 9F) with numerous distinctly modified black setae; ventral process of aedeagus (Figs 8C, 9H–J) with stout apical portion (in lateral view). Female genital segments: Fig. 9E. China: western Zhejiang (Fig. 1)	***Domene reitteri* Koch, 1939**
5	Elytra with more or less pronounced longitudinal ridges. Male sternite VIII with few modified setae at most, on either side of posterior excision with cluster of black setae; aedeagus with thinner ventral process in lateral view	**6**
–	Elytra without distinct longitudinal ridges. Chaetotaxy of male sternite VIII different; aedeagus with stouter ventral process in lateral or ventral (*Domene procera*) view	**8**
6	Posterior margin of abdominal tergite VII without palisade fringe. Male sternite VII (Assing and Feldmann 2014: figure 23) with weakly and broadly concave posterior margin; aedeagus (Assing and Feldmann 2014: figures 25–26) with longer ventral process. China: western Yunnan (Fig. 1)	***Domene immarginata* Assing & Feldmann, 2014**
–	Posterior margin of abdominal tergite VII with palisade fringe. Male sternite VII of different shape; aedeagus with shorter ventral process. Species from Taiwan	**7**
7	Legs yellowish brown to reddish; antennae brown to dark brown. Male sternite VIII (Assing and Feldmann 2014: figure 7) with deeper and slightly narrower posterior excision; ventral process of aedeagus (Assing and Feldmann 2014: figure 8) weakly curved in lateral view. Central western Taiwan: Taichung Hsien: Anma Shan	***Domene scabripennis* Rougemont, 1995**
–	Legs blackish-brown; antennae dark-brown to blackish-brown. Male sternite VIII (Assing and Feldmann 2014: figure 15) with shallower and broader posterior excision; ventral process of aedeagus (Assing and Feldmann 2014: figures 16–17) nearly straight in lateral view. Southern Taiwan: Kaohsiung Hsien	***Domene alesiana* Assing & Feldmann, 2014**
8	Habitus broader; head somewhat broader than pronotum. Punctation of head and pronotum coarser and less dense, surface therefore more shiny. Male sternite VIII with deeply and broadly U-shaped posterior excision, on either side of posterior excision with short, dense and dark peg-setae. Aedeagus (Coiffait 1982: figures 95, A–C). Russia: East Siberia, Far East; “Korea”; China: Northeast Territory	***Domene procera* Eppelsheim, 1886**
–	Habitus more slender; head about as broad as pronotum. Punctation of head and pronotum finer and denser, rendering them more matt. Male sternite VIII with V-shaped posterior excision, on either side of posterior excision with cluster of dense dark setae	**9**
9	Coloration of body (Fig. 5) black. On average smaller species (FL: 4.70–5.20 mm). Male sternite VIII (Fig. 6G) with deeper posterior excision; aedeagus (Figs 6H–J) smaller (1.07–1.18 mm). Northeastern Myanmar; China: western Yunnan (Fig. 1)	***Domene malaisei* Scheerpeltz, 1965**
–	Coloration of body dark brown. On average larger species (FL: 5.12–5.50 mm). Male sternite VIII with less deep posterior excision; aedeagus larger (> 1.30 mm)	**10**
10	Aedeagus (Figs 14H–J) larger (1.52–1.54 mm) and with longer, more slender ventral process. Female genital segments (Fig. 14E) with small symmetric, weakly sclerotized structure. China: central Sichuan (Fig. 1)	***Domene reducta* sp. n.**
–	Aedeagus smaller (< 1.48 mm) and with shorter, less slender ventral process. Female genital segments with asymmetric, moderately sclerotized structure	**11**
11	Punctation of elytra coarser and with more rugose interstices, surface nearly matt. Male sternite VII (Fig. 12F) with broadly concave posterior margin; male sternite VIII (Fig. 12G) with shallower impression; ventral process of aedeagus (Figs 12H–J) with less slender and less acute apical portion. Female genital segments (Fig. 12E) with smaller sclerotized structures. China: Gansu, Hubei, Shaanxi (Fig. 1)	***Domene cultrata* sp. n.**
–	Punctation of elytra less coarse and with less rugose interstices, surface slightly more shiny. Posterior margin of male sternite VII (Fig. 13F) concave in the middle; male sternite VIII (Fig. 13G) with deeper impression; ventral process of aedeagus (Figs 13H–J) with more slender and more acute apical portion. Female genital segments (Fig. 13E) with larger sclerotized structure. China: Gansu, Shaanxi, Sichuan (Fig. 1)	***Domene cuspidata* sp. n.**

## Supplementary Material

XML Treatment for Domene (Macromene) chenpengi

XML Treatment for Domene (Macromene) crassicornis

XML Treatment for Domene (Macromene) curtipennis

XML Treatment for Domene (Macromene) firmicornis

XML Treatment for Domene (Macromene) malaisei

XML Treatment for Domene (Macromene) reitteri

XML Treatment for Domene (Macromene) chenae

XML Treatment for Domene (Macromene) cultrata

XML Treatment for Domene (Macromene) cuspidata

XML Treatment for Domene (Macromene) reducta

XML Treatment for Domene (Macromene) sp.
